# The impact of comorbidity on mortality in Danish sarcoma patients from 2000-2013: A nationwide population-based multicentre study

**DOI:** 10.1371/journal.pone.0198933

**Published:** 2018-06-11

**Authors:** Mathias Raedkjaer, Katja Maretty-Kongstad, Thomas Baad-Hansen, Peter Holmberg Jørgensen, Akmal Safwat, Peter Vedsted, Michael Mørk Petersen, Thea Hovgaard, Tine Nymark, Johnny Keller

**Affiliations:** 1 Department of Orthopaedic Surgery, Aarhus University Hospital, Aarhus, Denmark; 2 Department of Orthopaedic Surgery, Rigshospitalet, University of Copenhagen, Copenhagen, Denmark; 3 Department of Experimental Clinical Oncology, Aarhus University Hospital, Aarhus, Denmark; 4 Department of Pathology, Aarhus University Hospital, Aarhus, Denmark; 5 Department of Oncology, Aarhus University Hospital, Aarhus, Denmark; 6 The Research Unit of General Practice, Aarhus University, Aarhus, Denmark; 7 Silkeborg Hospital, Department of Clinical Medicine, Aarhus University, Aarhus, Denmark; 8 Department of Orthopaedic Surgery, Odense University Hospital, Odense, Denmark; University of South Alabama Mitchell Cancer Institute, UNITED STATES

## Abstract

**Introduction:**

Sarcoma is a rare type of cancer. The incidence increases with age and elderly patients may have comorbidity that affects the prognosis. The aim of this study was to describe the type and prevalence of comorbidity in a nationwide population-based study in Denmark from 2000–2013 and to analyse the impact of the different comorbidities on mortality.

**Material and methods:**

The Danish Sarcoma Registry is a national clinical database containing all patients with sarcoma in the extremities or trunk wall from 2000 and onwards. By linking data to other registries, we were able to get patient information on an individual level including date and cause of death as well as the comorbidity type up to 10 years prior to the sarcoma diagnosis. Based on diseases in the Charlson Comorbidity Index, we pooled the patients into six categories: no comorbidity, cardiopulmonary disease, gastrointestinal disease, neurovascular disease, malignant neoplasms, and miscellaneous (diabetes, renal and connective tissue diseases). 2167 patients were included.

**Results:**

The prevalence of comorbidity was 20%. For patients with localized disease, comorbidity increased the disease-specific mortality significantly (HR 1.70 (95% CI 1.36–2.13)). For patients with metastatic disease at the time of diagnosis, comorbidity did not affect the disease-specific mortality (HR 1.05 (95% CI 0.78–1.42)). The presence of another cancer diagnosis within 10 years prior to the sarcoma diagnosis was the only significant independent prognostic factor of disease-specific mortality with an increase of 66% in mortality rate compared to patients with no comorbidity (HR 1,66 (95% CI 1.22–2.25)).

**Conclusion:**

Comorbidity is a strong independent prognostic factor of mortality in patients with localized disease. This study emphasizes the need for optimizing the general health of comorbid patients in order to achieve a survival benefit from treatment of patients with localized disease, as this is potentially modifiable.

## Introduction

Sarcoma is a rare cancer that accounts for approximately 1% of newly diagnosed cancers in Denmark [1,2]. Sarcomas occur in all age groups although the incidence increases with age [3–5]. Due to a demographic shift towards a larger elderly population, more patients will require treatment and follow-up in the future [6]. With increasing age, comorbidity will also be more frequent and may affect prognosis in several ways: as an independent cause of death, by delaying diagnosis, causing complications of treatment, or being the reason for less comprehensive or aggressive treatment [6].

To improve the prognosis, additional knowledge of the sarcoma prognosis is needed, which takes comorbidity into consideration. Studies have shown poorer survival among cancer patients with comorbidity [7–14]. In general, the 5-year survival rates have improved among cancer patients without comorbidity, but not among patients with severe comorbidity [12,15]. A recent single centre study from our group on soft tissue sarcoma patients, found that 25% of the patients had comorbidity [16]. Presence of comorbidity was associated with significantly increased overall and disease-specific mortality compared to soft tissue sarcoma patients without comorbidity [16]. However, the study was limited to only soft tissue sarcoma patients in a 30 year time period, and there has been substantial progress in diagnosis, treatment and pathology, thus more comprehensive studies are needed [17].

The aim of this study was to describe the type and prevalence of comorbidity in sarcoma patients in a Danish nationwide, multi-centre, population-based study, in a recent period of time (2000–2013), and furthermore to analyse the impact of the different comorbidities on mortality.

## Material and methods

The Danish population is 5.75 million [18]. All Danish residents are assigned a unique personal identification number (CPR number) and this facilitates the possibility of linking the patient to all of the Danish registers [19]. Since 2011, treatment of sarcomas in Denmark has been centralized in two centres in Aarhus and Copenhagen respectively. Until 2011, a centre for treatment of soft tissue sarcoma also existed at Odense University Hospital. All patients have been diagnosed and treated according to national guidelines by an experienced multidisciplinary sarcoma team [20,21].

### Data sources

The Danish Sarcoma Registry (DSR) is a national clinical database, administered by the Danish Sarcoma Group and supported by the Danish Regions [22]. All Danish patients with a newly diagnosed sarcoma in the extremities or trunk wall have been systematically registered from January 1, 2009. Patients from 2000–2008 have recently been added based on a validation through the Danish Cancer Registry (DCR) and the Danish National Pathology Registry [23,24]. DSR contains patient characteristics and detailed data on tumour characteristics, treatment, local and distant recurrences as well as death.

Information on comorbidities was obtained from the National Patient Registry (NPR) [25]. Since 1977, the NPR has recorded information on all hospital contacts including surgery, pathology and diagnostic codes, as well as hospitalisation and discharge dates on an individual level [26–28]. NPR covers more than 99% of all Danish hospital admissions [28]. Diagnoses are classified based on the Danish version of the WHO International Classification of Diseases (ICD); version 8 before 1994 and version 10 after.

Information on vital status was obtained from the Civil Registration System (CRS) established in 1968. CRS is updated on a daily basis and contains CPR number, date of birth, vital status, date of death or emigration for all residents of Denmark [19].

Causes of death were primarily accessed from the DSR and secondarily from the Cause of Death Registry (CDR). The CDR was established in 1973 and registration is mandatory by law. The registry contains information on the immediate and underlying causes of death on an individual level [29].

By linking data from the DSR, the NPR, the CDR and the CRS we got information on age, vital status, tumour information, patient characteristics (gender, age at diagnosis), treatment (surgery, margin, radiation, chemotherapy), relapse, date and cause of death, and comorbidity.

### Study population

A total of 3167 patients were treated at a Sarcoma Centre in Denmark between 2000–2013. We included patients with sarcomas located in the extremities or trunk. Due to biological variation patients aged 14 or less; borderline tumours; specific tumour subtypes, e.g., subcutaneous low-grade liposarcoma/atypical lipomatous tumour; gastrointestinal stromal tumours (GIST); Kaposis sarcoma; dermatofibrosarcoma protuberans; giant cell tumour; aggressive fibromatosis and atypical fibroxanthoma were excluded. Thus, 2167 patients were included in the present study.

### Comorbidity

Comorbidity was originally defined as any additional clinical event that existed or occurred during the clinical course of a patient with an index disease during the study period [30]. However, in the present study, we only considered comorbidity prior to the diagnosis. To classify the presence of comorbidity, we used the Charlson Comorbidity Index (CCI) [31]. It is based on 19 different medical conditions, each weighted and assigned 1–6 points according to its potential impact on mortality, derived from the relative risk estimates [31].

All discharge diagnoses included in the CCI were extracted from NPR for a period of 10 years prior to the date of the sarcoma diagnosis. Based on these, a CCI score was computed for each patient. Tumours in soft tissue and bone (ICD-8; 170, 171, 192.49–99 and ICD-10; C40-41, C47, C49) were not included. All discharge diagnoses of 30 days and all cancer diagnoses of 90 days prior to the sarcoma diagnosis were excluded to eliminate surveillance bias and diagnoses related to the sarcoma. The comorbidity level based on the CCI score was divided into three groups: No comorbidity (score 0), mild comorbidity (score 1–2), and moderate/severe comorbidity (score ≥3).

Due to a low number of patients in many of the 19 CCI groups and thus insufficient statistical precision, we were not able to perform statistical analyses for each of the registered diseases. Therefore, we merged the diagnoses from the original CCI into six comorbidity categories based on anatomical origin or organ system. We did not integrate the original CCI score in these categories. The six categories were ‘No comorbidity’, ‘Cardiopulmonary disease’, ‘Gastrointestinal disease’, ‘Neurovascular disease’, ‘Malignant neoplasms’, and ‘Miscellaneous diseases’ (diabetes with or without end organ damage, connective tissue disease and moderate/severe renal disease). The six comorbidity categories are shown in Table 1 and all corresponding ICD-8 and ICD-10-codes are available in S1 Table.

**Table 1:** Charlson comorbidity index, number of patients in each comorbidity category and matching scores for the 19 medical conditions.

Comorbidity Categories:	CCI score:	N (%)
**No Comorbidity**	0	1.741 (80.3)
**Cardiopulmonary disease:**		60 (2.3)
Myocardial infarction	1	
Congestive heart failure	1	
Chronic pulmonary disease	1	
**Gastrointestinal disease:**		18 (0.8)
Ulcer disease	1	
Mild liver disease	1	
Moderate/severe liver disease	3	
**Neurovascular disease:**		57 (2.6)
Peripheral vascular disease	1	
Cerebrovascular disease	1	
Dementia	1	
Hemiplegia	2	
**Malignant neoplasms:**		196 (9.0)
Any tumour *	2	
Leukaemia	2	
Lymphoma	2	
Metastatic solid tumour	6	
**Miscellaneous diseases:**		95 (4.4)
Connective tissue disease	1	
Diabetes	1	
Moderate/severe renal disease	2	
Diabetes with end organ damage	2	
AIDS	6	

CCI: Charlson Comorbidity Index; AIDS: acquired immunodeficiency syndrome.

* Excluding tumours in soft tissue and bone (iCD-8; 170, 171, 192.49–99 and iCD-10; C40-C41, C47, C49).

### Mortality

Mortality was assessed using overall and disease-specific mortality. Follow-up time was from the date of their sarcoma diagnosis until death, emigration, or end of study period (June 22, 2016). Disease-specific mortality was defined as death from sarcoma or death with known metastatic disease (ICD-8; 170, 171, 192.49–99 and ICD-10; C40-41, C47, C49).

### Statistical analysis

Descriptive statistics were used for the prevalence of comorbidity. Chi-squared test was used to compare the distribution of comorbidity between patient and tumour characteristics. In order to minimize bias we used causal directed acyclic graphs (DAGs) (S1 Fig) to select relevant covariates for the multiple regression model [32]. We adjusted for age, which is highly associated to comorbidity and a strong predictor of mortality [7–14]. Overall mortality was estimated using the Kaplan-Meier method. A Cox proportional hazard model was used to compute crude and adjusted hazard ratios (HR) for overall mortality. Because of dependent events, cumulative incidence was estimated for disease-specific mortality, treating death of other causes than sarcoma as a competing risk [33]. Multivariate competing risk regression analyses were used for both crude and adjusted disease-specific mortality [34]. Patients with ‘No comorbidity’ served as the reference group in both analyses. Effect modification was tested using likelihood ratio test. All test were two-sided and a p-value <0.05 was considered statistically significant. Estimates were made with corresponding 95% confidence intervals (CI). All analyses were performed in STATA 13.1 software.

### Ethics

The Danish Data Protection Agency (j.nr: 1-16-02-245-14), Statens Serum Institute (FSEID-1729) and the Danish Clinical Registries (j.nr: DSD-2017-03-02) approved this study.

## Results

### Patient characteristics and prevalence of comorbidity

The characteristics of the 2167 included patients are shown in Table 2 in relation to the CCI-score and in relation to the comorbidity categories. Of these, 1906 (88%) had localized disease at time of diagnosis. The median age at diagnosis was 56 years and 55% of the patients were men. Median follow-up time was 5.5 years (range 0.0–16.1 years). Most patients (88%) received surgery, 20% received radiotherapy and 12% chemotherapy as part of primary treatment. In total, 426 (20%) patients had registered comorbidity at the time of diagnosis (19% of patients with localized disease and 28% of patients with metastases). Moderate/severe comorbidity was found in 275 (13%) patients, mild comorbidity in 151 (7%) patients, and no comorbidity in 1741 (80%) patients. The largest comorbidity category was malignant neoplasms with 196 (9%) patients. The group of miscellaneous diseases consisted of 95 patients, where 65% had diabetes with or without end organ damage, 18% connective tissue disease and 16% moderate/severe renal disease. Table 1 shows the distribution of patients for each comorbidity category.

**Table 2:** Patient characteristics (N: 2167) by Charlson comorbidity index score and by comorbidity categories.

	N (%)	Charlson Comorbidity Index Score N (%)			Comorbidity Categories N(%)				
		0	1–2	≥3	Cardiopulmonary	Gastrointestinal	Neurovascular	Mal. neoplasms	Miscellaneous
Total	2167 (100)	1741 (80)	151 (7)	275 (13)	60 (3)	18 (1)	57 (3)	196 (9)	95 (4)
Age (years)									
Median (range)	56 (15–96)	54 (15–96)	64 (17–93)	66 (16–93)	61 (12–82)	71 (43–93)	68 (19–93)	54 (16–91)	66 (26–90)
15–49	759 (35)	697 (40)	21 (14)	41 (15)	11 (18)	1 (6)	5 (9)	32 (16)	13 (14)
50–69	823 (38)	648 (37)	66 (44)	109 (40)	25 (42)	7 (39)	23 (40)	82 (42)	38 (40)
≥70	584 (27)	395 (23)	64 (42)	125 (45)	24 (40)	10 (56)	29 (51)	82 (42)	44 (46)
Sex									
Female	972 (45)	776 (45)	63 (42)	133 (48)	20 (33)	8 (44)	21 (37)	102 (52)	45 (47)
Male	1195 (55)	965 (55)	88 (58)	142 (52)	40 (67)	10 (56)	36 (63)	94 (48)	50 (53)
Stage at diagnosis									
Localized	1906 (88)	1553 (89)	132 (87)	221 (80)	54 (90)	16 (89)	48 (84)	154 (79)	81 (85)
Metastatic	261 (12)	188 (11)	19 (13)	54 (20)	6 (10)	2 (11)	9 (16)	42 (21)	14 (15)
Tumour size (cm)									
Median (range)	8.9 (1–52)	9.0 (1–52)	8.7 (1–40)	8.4 (1–30)	7.8 (1–25)	7.9 (1–30)	8.8 (1–40)	8.6 (1–29)	8.6 (1–25)
Location									
Subcutaneous	630 (33)	510 (33)	41 (32)	79 (35)	15 (29)	6 (38)	19 (37)	49 (31)	31 (40)
Subfascial	1261 (67)	1027 (67)	87 (68)	147 (65)	37 (71)	10 (63)	33 (63)	107 (68)	47 (60)
Malignancy grade									
Low	509 (26)	434 (27)	29 (21)	46 (19)	11 (20)	1 (8)	13 (24)	31 (18)	19 (22)
Intermediate	399 (20)	325 (20)	31 (22)	43 (18)	11 (20)	5 (38)	10 (19)	31 (18)	17 (20)
High	1083 (54)	853 (53)	79 (57)	151 (63)	34 (60)	7 (54)	31 (57)	107 (63)	51 (59)
Treatment									
Surgery	1913 (88)	1555 (89)	128 (85)	230 (84)	50 (83)	13 (72)	50 (88)	161 (82)	84 (88)
Wide/radical	993 (41)	817 (60)	63 (57)	113 (56)	27 (60)	7 (70)	24 (57)	78 (57)	40 (51)
Intralesional/marginal	689 (59)	551 (40)	48 (43)	90 (44)	18 (40)	3 (30)	18 (43)	60 (43)	39 (49)
Radiotherapy	429 (20)	345 (20)	32 (21)	52 (19)	16 (27)	4 (22)	8 (14)	40 (20)	16 (17)
Chemoterapy	264 (12)	218 (13)	15 (10)	31 (11)	7 (12)	1 (6)	3 (5)	26 (13)	9 (9)

Notes: N: number. Mal.: Malignant. I Tumour size: 309 missing. Grade: 176 missing.

### Overall mortality

**Fig 1:**
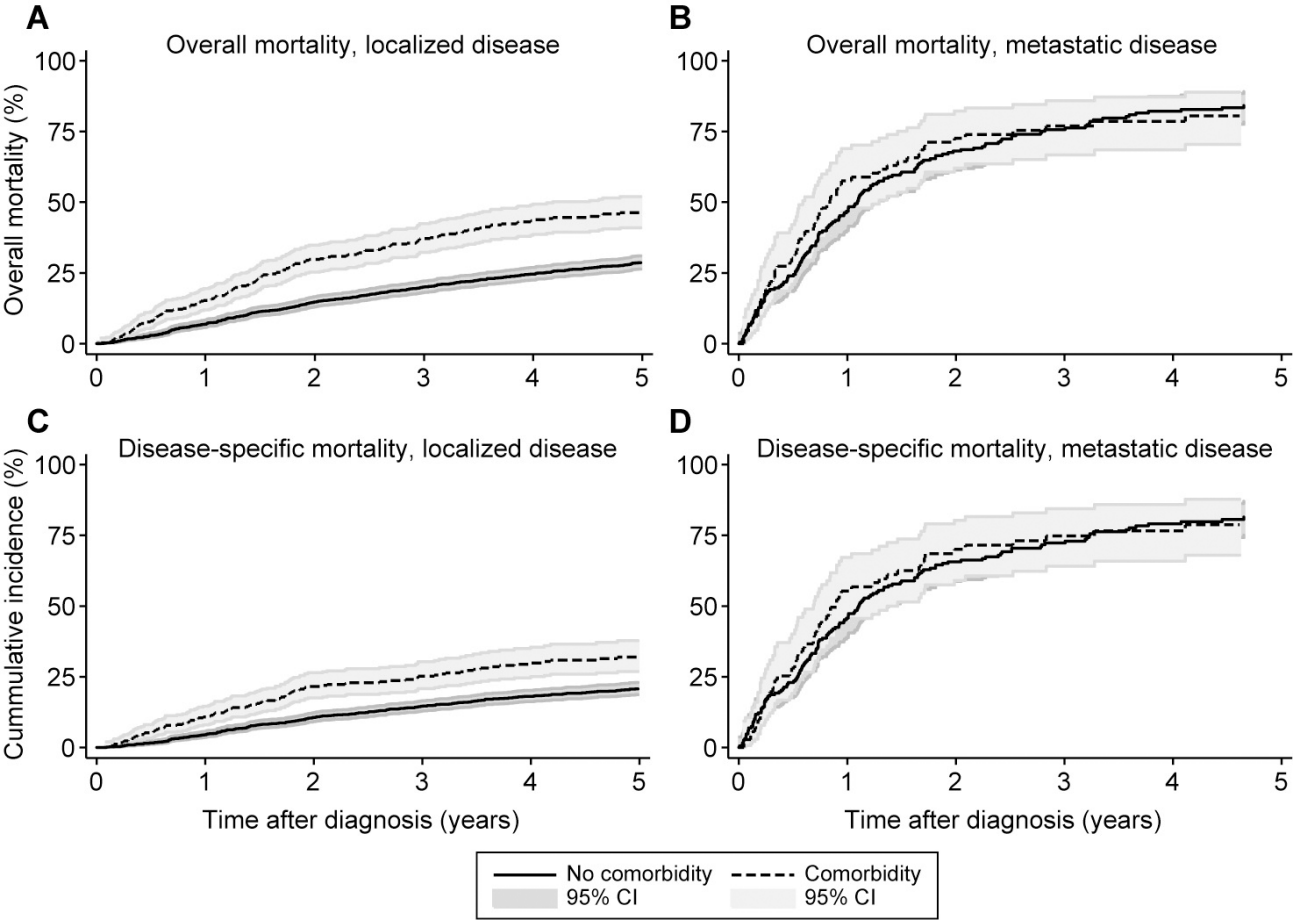
Estimates of impact of comorbidity on overall mortality and disease-specific mortality. Kaplan-Meier estimates of the impact of comorbidity on overall mortality for patients with (A) localized disease and (B) metastatic disease. Cumulative incidence curves of the impact of comorbidity in disease-specific mortality in (C) localized disease and (D) metastatic disease.

For patients with localized disease at the time of diagnosis, the one- and five-year overall mortality was 8% (95% CI 7–10) and 32% (95% CI 30–34), respectively. For patients with metastatic disease, the one- and five-year overall mortality was 50% (95% CI 44–56) and 83% (95% CI 30–34), respectively. The overall mortality for comorbidity vs. no comorbidity is shown in Fig 1A and 1B.

Mortality significantly increased in patients with comorbidity and localized disease at the time of diagnosis, with a crude HR of 1.89 (95% CI 1.59–2.25) compared to patients without comorbidity. Mortality did not differ for comorbidity vs. no comorbidity in patients with metastatic disease at the time of diagnosis (crude HR: 1.05 (95% CI 0.78–1.42)).

As seen in Table 3, the adjusted overall mortality was significantly higher in the comorbidity categories gastrointestinal disease, malignant neoplasms, and miscellaneous diseases.

**Table 3:** Crude and adjusted analyses for the association between type of comorbidity, and the overall and disease-specific mortality in patients with localized and metastatic disease respectively (n = 2167).

		Overall mortality				Disease-specific mortality			
				HR (95% CI)				HR (95% CI)	
	N	1-year (%)	5-year (%)	Crude	Adjusted*	1-year (%)	5-year (%)	Crude	Adjusted*
Total	2167								
**Localized**									
Overall	1906	8	32			6	23		
No comorbidity	1553	7	29	1	1	4	20	1	1
Cardiopulmonary	54	13	39	1.48 (0.97–2.25)	1.21 (0.80–1.84)	6	26	1.52 (0.91–2.55)	1.36 (0.81–2.28)
Gastrointestinal	16	31	68	3.14 (1.72–5.70)	1.86 (1.02–3.40)	13	34	2.11 (0.87–2.07)	1.64 (0.67–3.98)
Neurovascular	18	13	49	1.99 (1.31–3.02)	1.33 (0.87–2.02)	6	20	1.02 (0.51–2.07)	0.84 (0.41–1.70)
Malignant neoplasm	154	16	45	1.83 (1.44–2.34)	1.41 (1.10–1.80)	13	31	1.90 (1.41–2.57)	1.66 (1.22–2.25)
Miscellaneous**	81	14	48	2.85 (1.50–2.81)	1.48 (1.07–2.03)	11	31	1.78 (1.17–2.69)	1.51 (0.99–2.30)
**Metastatic**									
Overall	261	50	83			48	81		
No comorbidity	188	47	84	1	1	45	78	1	1
Cardiopulmonary	6	50	-	0.84 (0.31–2.26)	0.69 (0.25–1.86)	50	71	0.91 (0.34–2.46)	0.75 (0.27–2.03)
Gastrointestinal	2	50	-	1.16 (0.29–4.69)	0.76 (0.18–3.26)	-	60	0.63 (0.09–4.53)	0.43 (0.06–3.20)
Neurovascular	9	78	78	1.61 (0.76–3.45)	1.23 (0.57–2.66)	67	66	1.50 (0.66–3.40)	1.15 (0.50–2.64)
Malignant neoplasm	42	60	79	1.01 (0.70–1.47)	0.82 (0.56–1.22)	57	76	1.07 (0.73–1.56)	0.87 (0.59–1.30)
Miscellaneous**	14	43	-	1.02 (0.57–1.84)	0.91 (0.51–1.65)	43	80	1.01 (0.55–1.86)	0.90 (0.49–1.67)

CI: confidence interval; HR: hazard ratio; N: number.

* Analyses adjusted for age.

** Diabetes with or without end organ damage, connective tissue disease, and moderate/severe renal disease

There was no association between type of comorbidity and mortality for patients with metastatic disease at time of diagnosis.

### Disease-specific mortality

A total of 631 (29%) patients died from sarcoma. For patients with localized disease, the one- and five-year disease-specific mortality was 6% (95% CI 5–7) and 23% (95% CI 21–25), respectively. For patients with metastatic disease, the one- and five-year disease-specific mortality was 48% (95% CI 42–55) and 81% (95% CI 75–86), respectively. Cumulative incidence curves of disease-specific mortality are shown in Fig 1C and 1D. For patients with localized disease, disease-specific mortality significantly increased in the presence of comorbidity (70% increase in mortality rate (95% CI 1.36–2.13)). For patients with metastatic disease at the time of diagnosis, comorbidity did not affect the disease-specific mortality (5% increase in mortality rate (95% CI 0.78–1.42)). The crude disease-specific mortality was significantly higher in the comorbidity categories malignant neoplasms (90% increase in mortality rate (95% CI 1.41–2.57)) and miscellaneous diseases (78% increase in mortality rate (95% CI 1.17–2.69)), as seen in Table 3. Adjusting for age, the presence of another cancer diagnosis within ten years prior to the sarcoma diagnosis was the only significant independent prognostic factor of disease-specific mortality with a 66% increase in mortality level (95% CI 1.22–2.25) compared to patients with no comorbidity.

Among patients with metastatic disease at the time of diagnosis, there was no significant difference in disease-specific mortality for different comorbidity categories.

## Discussion

In this nationwide, population-based study of 2167 sarcoma patients, we found an overall prevalence of comorbidity of 20% based on the CCI. The level of comorbidity had a significant negative impact in both overall and disease-specific mortality in patients with localized disease at the time of diagnosis. This suggests that the survival is modifiable in the group of patients with comorbidity and that an early intervention could change the prognosis. Especially among those with a previous cancer diagnosis, who had a 66% greater risk of dying from their sarcoma than patients without comorbidity. No impact of comorbidity on mortality was found in patients with metastatic sarcoma.

### Methodological considerations

The strengths of this study include the population-based design, the use of reliable national registries, and the homogeneous healthcare provided in Denmark. We had a relatively large sample size in a limited time period, and long-term follow-up. This ensured a high statistical precision.

The DSR contains data on treatment, follow-up, and vital status for all sarcoma patients in Denmark, which prevented selection bias. The study included all patients treated in a defined and recent time period and thus the group of patients can be considered homogenous regarding the diagnostic evaluation process and treatment. Information bias from the administrative registries NPR, CDR, and CRS is low. Still, the data in the national registries depends on the correctness of the physicians when the medical records are filled in. The comorbidity diagnoses were recorded in a 10-year period before the sarcoma diagnosis, and thus, any misclassification is non-differential. A review of 950 medical records to verify the NPR diagnoses using ICD-10 codes found a positive predictive value of 98% for the CCI conditions [35].

The limitations of this study include the risk of differential misclassification of the cause of death by using registries such as the CDR. If the patient dies in close temporal proximity to the sarcoma diagnosis or treatment; there will be an inclination to state the sarcoma as cause of death.

Using the CCI to assess the level of comorbidity in cancers is common, and has been validated several times [8,11,13,36]. Only hospital-based diagnoses of comorbidity were registered in this study. Thus, there is a probability of underestimating some of the mild conditions in the NPR, as these are mainly treated in primary care.

The comorbidities were categorised based on anatomical origin or affected organ system. Both mild and severe diseases were pooled in the same comorbidity categories. This may have lowered the detailed information about the specific comorbidities in question, but the statistical precision increased considerably. We pooled patients with both soft tissue and bone sarcomas in relation to comorbidity and mortality, although the variation in biological manners, distribution of age, and treatment is different to some extend. Finally, no data on life style factors such as smoking, body mass index, and use of alcohol or medication was provided, all of which factors that possibly affects the mortality rate.

### Comparison with other studies

The 20% prevalence of comorbidity in this nationwide study is in concordance with the few previous studies in sarcoma patients, which found a 19% prevalence of comorbidity in bone sarcoma patients and 25% of soft tissue sarcoma patients [16,37]. These studies were single centre retrospective studies with a span of 30 years. 5-year disease-specific mortality was 26% for soft tissue sarcoma patients without comorbidity and 33% to 44% for patients with mild to severe comorbidity. Compared to our findings there is a considerable difference in disease-specific survival both in the group of patients with no comorbidity as well as patients with comorbidity. This could be due to the extensive progress in diagnosis and treatment and advancements in diagnostic imagining and evaluation of surgical materials the last decades. Greater accessibility to CT scan, the emergence of MRI scan and the increased use of ultrasound might have diminished the diagnostic window [17]. New forms of chemotherapy, progress in surgical techniques including focus on wide resection, and the implementation of the urgent fast-track referral pathway in Denmark in January 2009 could all be underlying reasons for improved outcome [17,38]. Furthermore, in this present study we found that gastrointestinal disease, malignant neoplasms, and miscellaneous diseases had significant impact on overall mortality, while a preceding cancer diagnose had significantly impact on disease-specific mortality. A sarcoma-specific comorbidity index was the ideal outcome of this study in order to identify specific diseases, but the representation of the different 19 diseases in the study population was unfortunately not large enough. Still, the comorbidity categories might imply where special attention should be made in order to improve the outcome for sarcoma patients with comorbidity. Other studies have reported a prevalence of comorbidity in soft tissue sarcoma patients at 12%; however, this was at the time of diagnosis, and did not include discharge diagnoses in a 10-year period before diagnosis [39].

Several studies have investigated the prevalence of comorbidity according to the CCI in other cancers such as prostate (37%), ovarian (24%), colorectal (14–34%), breast (13–20%), lung (50%), and head and neck cancer (33%) [9,11,12,14,40–42]. In general, the proportion of patients with comorbidity is greater in other cancers. This could be due to a relatively low median age of 56 years and a considerable group of younger patients among the bone sarcomas [39]. It could also be due to aetiological factors, e.g., smoking in lung cancer and smoking and alcohol abuse in head and neck cancer [9,11].

The prevalence of comorbidity in sarcoma patients seems to reflect the comorbidity of the general population. This is supported by studies, where prevalence of comorbidity according to the 19 CCI conditions in soft tissue sarcoma patients and an age- and sex-matched cohort was comparable, except for ‘any tumour’ and ‘metastatic solid tumour’ [43]. According to a UK retrospective study of 287,459 primary care patients, 26.5% of the patients had comorbidity according to the diagnoses in the CCI. The prevalence of comorbidity was 14% as calculated from the diagnoses within the CCI in 21,868 age-matched controls in a study with 5,192 breast, lung, colon, prostate and ovarian cancer patients [44].

We found that the presence of comorbidity had a significant impact on both overall and disease-specific mortality in patients with localized disease at the time of diagnosis. This is in concordance with previous findings in studies of soft tissue sarcomas [16]. Studies in bone sarcomas have shown that comorbidity was a strong independent prognostic factor on overall survival but not on disease-specific mortality [37]. The impact of comorbidity on cancer survival has been investigated in other cancers. In head and neck cancer, comorbidity was a strong independent prognostic factor on overall survival, but not for cancer-specific death [11]. Studies of breast cancer show that the presence of comorbidity had a strong independent effect on both disease-specific and overall survival [45,46].

In patients with metastatic disease at the time of diagnosis, we found that comorbidity did not have an impact on mortality. This is most probably due to the poorer prognosis of a metastatic cancer.

Comorbidity may mask early cancer symptoms leading to delay and therefore a more advanced stage of the disease at time of diagnosis. However, other studies have found a higher prevalence of comorbidity in early-stage cancers [9,45]. An explanation could be that patients receiving more frequent medical care due to their comorbidity are more likely to receive clinical monitoring.

Surgery with wide margins is the primary curative treatment of soft tissue sarcomas and bone sarcomas, alone or in a combination with radiotherapy and/or chemotherapy [5]. Certain comorbidities and associated medical treatments may serve as an absolute or relative contraindication for optimal treatment. This may affirm the influence of comorbidity on mortality as seen in our study.

## Conclusion

In this nationwide, population-based study, we found a prevalence of comorbidity of 20% of sarcoma patients. The comorbidity categories gastrointestinal disease, malignant neoplasms, and miscellaneous diseases had significant independent influence on overall mortality. Disease-specific mortality was affected only by a preceding cancer diagnose.

The presence of comorbidity was a strong independent prognostic factor on overall as well as disease-specific mortality in patients with localized disease but not among patients with metastases at the time of diagnosis. The study emphasizes the need for improving the general health of patients with comorbidity in order to gain a survival benefit from treatment of patients with localized disease, as this is potentially modifiable.

## Supporting information

S1 Table ICD-8 and ICD-10 codes used to calculate the Charlson Comorbidity Index and matching scores for the 19 medical conditions. The medical conditions are divided in comorbidity categories.
(PDF)

S1 Fig Directed acyclic graph of possible relationship between important covariates and mortality in sarcoma patients. (PDF)
