# Supplementary material for: The impact of comorbidity on mortality in Danish sarcoma patients from 2000-2013: A nationwide population-based multicentre study
Source: PLoS One. 2018 Jun 11;13(6):e0198933. doi: 10.1371/journal.pone.0198933 (PMC5995448; doi:10.1371/journal.pone.0198933)
Supplement: S1 Table — The medical conditions are divided in comorbidity categories. (PDF) [file pone.0198933.s001.pdf]

S1 table: ICD-8 and ICD-10 codes used to calculate the Charlson Comorbidity Index and matching scores for the 19 medical conditions. The medical conditions are divided in comorbidity categories.

Comorbidity category: Conditions:	ICD-8:	ICD-10:	CCI-Score:
**Cardiopulmonary disease:**			
Myocardial infarction	410	I21; I22; I23	1
Congestive heart failure	427.09; 427.10; 427.11; 427.19; 428.99; 782.49	I50; I11.0; I13.0; I13.2	1
Chronic pulmonary disease	490-493; 515-518	J40-J47; J60-J67; J68.4; J70.1; J70.3; J84.1; J92.0; J96.1; J98.2; J98.3	1
**Gastrointestinal disease:**			
Ulcer disease	530.91; 530.98; 531–534	K22.1; K25–K28	1
Mild liver disease	571; 573.01; 573.04	B18; K70.0–K70.3; K70.9; K71; K73; K74; K76.0	1
Moderate/severe liver disease	070.00; 070.02; 070.04; 070.06; 070.08; 456.00–456.09; 573.00	B15.0; B16.0; B16.2; B19.0; K70.4; K72; K76.6; I85	3
**Neurovascular disease:**			
Peripheral vascular disease	440–445	I70; I71; I72; I73; I74; I77	1
Cerebrovascular disease	430–438	I60–I69; G45; G46	1
Dementia	290.09–290.19; 293.09	F00–F03; F05.1; G30	1
Hemiplegia	344	G81; G82	2
**Malignant neoplasm:**			
Any tumour*	140–169; 172–192.48; 193–194	C00–C39; C42–C46; C48; C50–C75	2
Leukaemia	204–207	C91–C95	2
Lymphoma	200–203; 275.59	C81–C85; C88; C90; C96	2
Metastatic solid tumour	195–199	C76–C80	6
**Miscellaneous diseases:**			
Connective tissue disease	135.99; 446; 712; 716; 734	M05; M06; M08; M09; M30; M31; M32; M33; M34; M35; M36; D86	1
Diabetes	249.00; 249.06; 249.07; 249.09; 250.00; 250.06; 250.07; 250.09	E10.0, E10.1; E10.9; E11.0; E11.1; E11.9	1
Moderate/severe renal disease	403; 404; 580–583; 584; 590.09; 593.19; 753.10–753.19; 792	I12; I13; N00–N05; N07; N11; N14; N17–N19; Q61	2
Diabetes with end organ damage	249.01–249.05; 249.08; 250.01–250.05; 250.08	E10.2–E10.8; E11.2–E11.8	2
AIDS	079.83	B21–B24	6

ICD: international Classification of Disease (version 8 and 10); CCI: Charlson Comorbidity Index; AIDS: acquired immunodeficiency syndrome.

∗ Excluding tumours in soft tissue and bone (iCD-8; 170, 171, 192.49-99 and iCD-10; C40-C41, C47, C49).
