# Supplementary material for: The impact of comorbidity on mortality in Danish sarcoma patients from 2000-2013: A nationwide population-based multicentre study
Source: PLoS One. 2018 Jun 11;13(6):e0198933. doi: 10.1371/journal.pone.0198933 (PMC5995448; doi:10.1371/journal.pone.0198933)
Supplement: S1 Fig — (PDF) [file pone.0198933.s002.pdf]

S1 fig: Directed acyclic graph of possible relationship between important covariates and mortality in sarcoma patients.

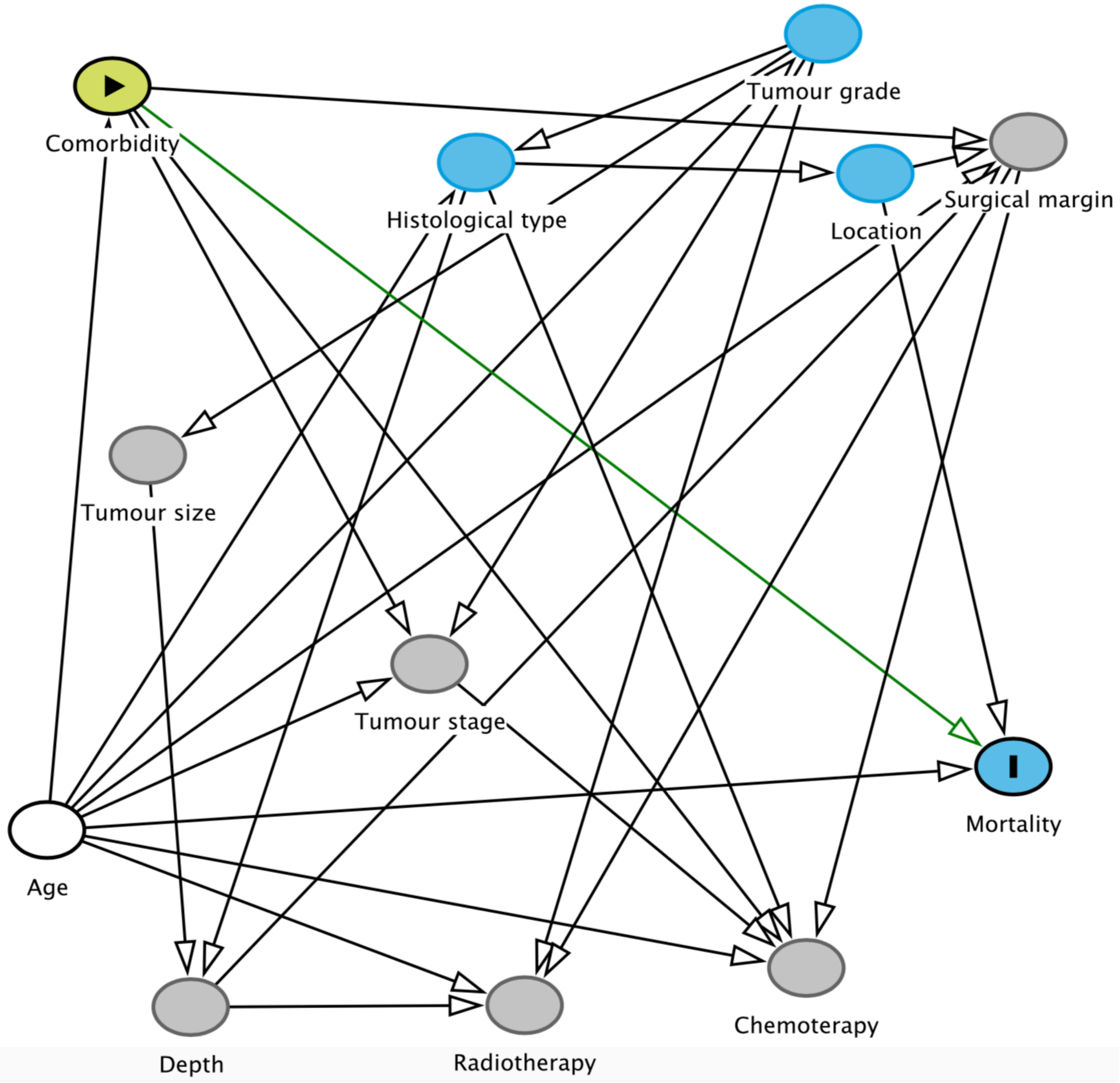

Exposure: Comorbidity
Outcome: Death
Adjusted variable: Age
